# Osteogenesis imperfecta and pregnancy: a case report

**DOI:** 10.1186/s13256-019-2296-0

**Published:** 2019-12-11

**Authors:** Felix Chamunyonga, Kudzaishe Lloyd Masendeke, Bismark Mateveke

**Affiliations:** Department of Obstetrics and Gynecology, University of Zimbabwe, College of Health Sciences, Harare, Zimbabwe

**Keywords:** Osteogenesis imperfecta, Pregnancy, Prenatal diagnosis

## Abstract

**Background:**

Osteogenesis imperfecta is a rare connective tissue disorder of varying phenotypic presentations. In pregnancies complicated by osteogenesis imperfecta, there is an increased risk to both the mother and fetus.

**Case presentation:**

We present a case of a 34-year-old, wheelchair-bound, primigravid African (Zimbabwean) patient with short stature and skeletal deformities. Her care, requiring a multidisciplinary team approach, resulted in the delivery of a live baby girl with a birth weight of 2100 g, also with osteogenesis imperfecta.

**Conclusion:**

Good outcomes are reported when a multidisciplinary team is involved in the care of patients with osteogenesis imperfecta. Pregnancies can be carried to term but require close antenatal surveillance. Prenatal diagnosis is possible with ultrasound and genetic testing. Delivery should be carefully planned by a multidisciplinary team. Decisions on delivery mode should be made on a case-by-case basis.

## Background

Osteogenesis imperfecta (OI), also known as *brittle bone disease*, is a phenotypically diverse disorder due to deficiencies in the synthesis of type I collagen [1]. OI is a disease characterized by brittle bones and frequent fractures with minimal trauma leading to skeletal deformities [2]. Its incidence is estimated at 1 per 20,000 births [3]. Though rare, it is the most common inherited disorder of connective tissue. Principally, it affects bone, but it also impacts other tissues rich in type I collagen, such as joints, eyes, ears, skin, and teeth. It usually results from an autosomal dominant mutation (over 800 identified) in the genes that code for alpha-1 and alpha-2 chains of collagen and involves the substitution of glycine residues in the triple-helical domain [2]. Location of the mutation within the protein determines the genotype-phenotype relationship underlying OI. There are nine major phenotypically different subtypes, which vary widely in severity. Types I to IV are the best defined [4].

Advances in medicine mean that more women affected by skeletal disorders survive into their reproductive years, desire fertility, and become pregnant. There is a need for serious consideration of pregnancy and preconception counseling. Undesired pregnancies should be avoided entirely. Pregnancy in OI poses a major life-threatening risk to both mother and child [5].

A woman with skeletal deformities such as kyphoscoliosis and severe chest wall deformities is at risk of cardiorespiratory complications. Severe backache and musculoskeletal pain can be severe enough to disrupt daily activity. Immobility increases the risk of venous thromboembolism. Impaired wound healing, increased risk of blood loss during delivery, and increased prevalence of cardiac abnormalities and congenital heart disease contribute to complications associated with OI [6].

With an autosomal dominant pattern, there is a 50% chance of a child of a woman with OI being affected. Historically diagnosed by plain radiographs, ultrasonography now allows diagnosis before 20 weeks of gestation [7]. Parents can also be offered invasive fetal diagnostic testing for OI, chorionic villi sampling, or amniocentesis with analysis for mutations of *COLIA1* on chromosome 16 and *COLIA2* on chromosome 7 [2]. It is necessary to provide genetic, maternal-fetal medicine, and neonatal consultation to assess and discuss the fetal risk of OI, implications of OI on pregnancy and delivery, and risk to the fetus.

## Case presentation

A 34-year-old African (Zimbabwean) woman in her first pregnancy presented to our institution at 22 weeks of gestation by referral from a local clinic with a diagnosis of OI. She was wheelchair-bound. Her medical history revealed several fractures as a neonate and in childhood, leading to a diagnosis of OI based on clinical presentation and examination. Confirmatory tests were not available in the public health sector. She has been in and out of the hospital since childhood because of these fractures, with various treatments having been received, including plaster of Paris casts and traction. She had a fracture of the right femur at the age of 12 years, which made her wheelchair-dependent. She can, however, stand with support and climb onto and off the wheelchair without assistance. She was abnormally short compared with her siblings and peers. She had normal pubertal development, and her menstrual cycle was very regular. Her sexual debut was at 28 years, and she has had one sexual partner. Her boyfriend, who was married and had five other children, contributed little to her welfare. She was a vendor receiving some financial support from her siblings. She took combined oral contraceptives for 4 years; however, she had stopped taking them because she thought she could not get pregnant. The result of cervical cancer screening with visual inspection with acetic acid and cervicography done in 2016 was negative. She had no family history suggestive of OI. She is educated to form 4 (ordinary level). This current pregnancy, though unplanned, was booked at 18 weeks of gestation. The results of antenatal screening for human immunodeficiency virus and syphilis were negative.

On examination, she was of short stature, 120 cm in height, with a triangular face. Her sclera was white. She had poor dentition with grayish discoloration. She had short limbs, with the right lower limb shorter than the left, and deformities were noted on both thighs. She did not have scoliosis or kyphosis. Her chest was barrel-shaped with good air entry bilaterally. She had normal blood pressure and a normal cardiovascular system examination finding. Her abdominal examination revealed central obesity and a bulky uterus of 20-week-size gestation.

A multidisciplinary team was involved in her management. The team included obstetricians, a maternal-fetal medicine specialist, pediatricians, anesthetists, a psychologist, and midwives. Ultrasound performed by a maternal-fetal medicine specialist showed a fetus with a bowed femur and short humerus. No fractures were noted. The fetal skull was easily deformable. No other malformations were noted. The conclusion was that the fetus had OI, nonsevere disease. Pediatricians counseled the patient about the fetal condition and the possibility of preterm delivery and its accompanying complications. With the limitations of the neonatal unit, the conclusion was an unfavorable prognosis for the neonatal outcome. The patient, however, was willing to continue with the pregnancy.

She was subsequently followed closely in the antenatal clinic, with a plan for pregnancy to continue to 37 weeks, with the possibility of early delivery if maternal respiratory compromise occurred. She had an uneventful antenatal period, with serial ultrasound showing satisfactory growth. She had an admission for a urinary tract infection at 29 weeks of gestation and was treated with oral antibiotics with complete resolution. Contraceptive counseling was provided, and she opted for tubal ligation, which was done at the time of cesarean section.

She ultimately delivered, by an elective transverse lower segment cesarean section at 37 weeks, a live female fetus with a birth weight of 2100 g. The operation was done with the patient under general anesthesia, and a smooth intubation was done with care so as not to cause cervical spinal fracture. Bilateral tubal ligation was performed. Estimated blood loss at the time of delivery was 500 ml, and 10 IU of oxytocin was administered for delivery of the placenta. The patient was extubated after surgery and admitted to the high-dependency unit and monitored for 24 hours. The baby was admitted to the neonatal unit. She developed a chest infection, which was successfully treated with intravenous antibiotics. The mother was managed for a paralytic ileus on day 2 after surgery. She was discharged on day 7. She developed a superficial surgical site infection, which was managed with daily dressings and oral antibiotics on an outpatient basis. The child is currently seen in follow-up by her pediatrician for OI. She has blue sclera. She was noted to have healed fractures on the left radius and right femur at 6 weeks of age. Initial neonatal x-rays had not revealed any fractures. Bone deformities were also noted. She can now walk with support. The rest of her developmental milestones are normal. No other fractures have been reported since they were originally noted. The parents are not able to afford genetic tests.

## Discussion

OI is a rare condition with sparse reports from Africa and also in black populations from other regions [7]. The main characteristic feature is brittle bones leading to repeated fractures after minimal trauma. Diagnosis is mainly clinical [7]. Radiological investigations and collagen or genetic analysis may be necessary in some cases. Results of routine laboratory studies are usually within normal ranges and are useful in ruling out other metabolic bone diseases.

A multidisciplinary approach is necessary when caring for a pregnant woman with OI. The team may include a perinatologist, obstetrician, pediatrician, anesthetists, orthopedists, geneticists, and surgeons, among other specialists [1].

Fertility is preserved, especially in mild forms of OI, and pregnancy, such as in our patient, can be carried to term. A good outcome has been reported in pregnant women with OI and their neonates [8]; however, it is dependent on the severity of each individual case. Essential components in the care of women with OI include a preconception assessment and close antenatal monitoring with an MDT approach.

Prenatal diagnosis by ultrasound is possible [7]. Ultrasound performed at 22 weeks of gestation by the maternal-fetal medicine specialist led to a probable diagnosis of OI, nonsevere disease, in our patient. It is, however, important to know that mild forms may not be evident on an ultrasound scan.

The antenatal period can be complicated by musculoskeletal problems. Common complications are Severe backache related to crush fracture of vertebrae; spinal deformity, disc, and ligament problems; and nonvertebral fracture. Respiratory compromise and discomfort in patients with chest deformities and small body habitus may necessitate preterm delivery in 27% of cases [8]. Our patient was able to reach term, when a planned cesarean delivery was executed.

Mode of delivery is chosen for the usual obstetric indications. One study showed a cesarean section rate of 54%. The indications included a nonvertex delivery in 53%, and an antenatal diagnosis of OI was made in less than 15%. There was a high rate of 37% of breech at term [9]. Cesarean section did not decrease the rate of fractures at birth in infants with nonlethal OI and did not prolong survival for those with lethal forms. The prenatal diagnosis did not influence the mode of delivery in most instances [9]. The indication for cesarean delivery in our patient was severe skeletal deformities and short stature.

Planning for the cesarean section involved a preoperative visit and planning by anesthetists. Spinal deformities make regional anesthesia difficult. Endotracheal anesthesia may be complicated by the risk of fracture to the jaw, cervical spine, and ribs. There might be delayed extubation due to respiratory muscle weakness in the late phase of surgical anesthesia. Need for intensive care postoperatively if cardiorespiratory function is compromised should be considered. Malignant hyperthermia is more common in individuals with OI [6]. In preparation, our patient underwent chest x-ray, echocardiography, and electrocardiogram, which were all reassuring. Intubation was potentially difficult due to fused cervical spinal bones, and the aim was a smooth intubation to avoid cervical fractures. The cesarean section was done with the patient under general anesthesia and was uneventful at both the anesthetic and surgical sites. She developed a paralytic ileus, however, which was managed conservatively, as well as a superficial surgical site infection, which resolved with dressings and antibiotics.

Neonates born with OI may have complications resulting from prematurity, with approximately 31% receiving neonatal intensive care unit (NICU) admission. There is a significant association of younger maternal age, preterm delivery, and NICU admission [8]. Fractures were present in 18% of neonates and bone deformity in 18%, and 22% had respiratory problems [8]. The baby of our patient was admitted to the NICU for low birth weight and close monitoring. She had a chest infection, which was treated with antibiotics. A skeletal survey x-ray revealed no fractures. She has recurrent chest infections, for which she is being followed by pediatricians.

Some studies have shown that bisphosphonates are an effective symptomatic treatment for children with OI [10]. Their use has been shown to improve bone mineral density and reduce fracture rates. Little is known about their effects on the developing fetus [11]. Bisphosphonates cross the placenta and have potential effects on developing bone. A review of 51 cases of preconception and antenatal bisphosphonate exposure has been reassuring and has shown no skeletal abnormalities or congenital malformations in the infants [12]. A study done in South Africa showed that treatment with bisphosphonates is well tolerated, associated symptomatic improvement, and highly rated by patients and parents [13]. That was the first study to look at the use of bisphosphonates in black South Africans. In our setting, no protocols are available for use of bisphosphonates before conception or for children with OI. They are also not available in the public health sector.

## Conclusions

Pregnant women with OI and pregnancy, with clinical teamwork and close follow-up, can carry a pregnancy to term successfully. Preconception counseling should be offered to those patients planning a pregnancy or to those who desire effective contraception, including sterilization for those who choose not to have a pregnancy or after a pregnancy.

## Data Availability

The data sets supporting the article are included within the article. Any additional information is available from the authors upon reasonable request.

## Abbreviations

NICU: Neonatal intensive care unit
OI: Osteogenesis imperfecta
